# Amino acid δ^15^N underestimation of cetacean trophic positions highlights limited understanding of isotopic fractionation in higher marine consumers

**DOI:** 10.1002/ece3.6142

**Published:** 2020-03-04

**Authors:** Cory J. D. Matthews, Rocio I. Ruiz‐Cooley, Corinne Pomerleau, Steven H. Ferguson

**Affiliations:** ^1^ Arctic Aquatic Research Division Fisheries and Oceans Canada Winnipeg MB Canada; ^2^ Departamento de Oceanografía Biológica Centro de Investigación Científica y de Educación Superior de Ensenada (CICESE) Ensenada México; ^3^ Moss Landing Marine Laboratories California State University Moss Landing CA USA; ^4^ Institut Maurice Lamontagne, Fisheries and Oceans Canada Mont‐Joli QC Canada; ^5^ Department of Biological Sciences University of Manitoba Winnipeg MB Canada

**Keywords:** amino acids, compound‐specific stable isotope analysis, CSIA‐AA, glutamic acid, nitrogen, phenylalanine, source, trophic discrimination factor

## Abstract

Compound‐specific stable isotope analysis (CSIA) of amino acids (AAs) has been rapidly incorporated in ecological studies to resolve consumer trophic position (TP). Differential ^15^N fractionation of “trophic” AAs, which undergo trophic ^15^N enrichment, and “source” AAs, which undergo minimal trophic ^15^N enrichment and serve as a proxy for primary producer δ^15^N values, allows for internal calibration of TP. Recent studies, however, have shown the difference between source and trophic AA δ^15^N values in higher marine consumers is less than predicted from empirical studies of invertebrates and fish. To evaluate CSIA‐AA for estimating TP of cetaceans, we compared source and trophic AA δ^15^N values of multiple tissues (skin, baleen, and dentine collagen) from five species representing a range of TPs: bowhead whales, beluga whales, short‐beaked common dolphins, sperm whales, and fish‐eating (FE) and marine mammal‐eating (MME) killer whale ecotypes. TP estimates (TP_CSIA_) using several empirically derived equations and trophic discrimination factors (TDFs) were 1–2.5 trophic steps lower than stomach content‐derived estimates (TP_SC_) for all species. Although TP_CSIA_ estimates using dual TDF equations were in better agreement with TP_SC_ estimates, our data do not support the application of universal or currently available dual TDFs to estimate cetacean TPs. Discrepancies were not simply due to inaccurate TDFs, however, because the difference between consumer glutamic acid/glutamine (Glx) and phenylalanine (Phe) δ^15^N values (δ^15^N_Glx‐Phe_) did not follow expected TP order. In contrast to pioneering studies on invertebrates and fish, our data suggest trophic ^15^N enrichment of Phe is not negligible and should be examined among the potential mechanisms driving “compressed” and variable δ^15^N_Glx‐Phe_ values at high TPs. We emphasize the need for controlled diet studies to understand mechanisms driving AA‐specific isotopic fractionation before widespread application of CSIA‐AA in ecological studies of cetaceans and other marine consumers.

## Graphical abstract

Low trophic position estimates using CSIA‐AA caution validation studies needed before widespread application to large marine consumers.

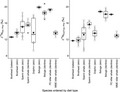

## 1 INTRODUCTION

Trophic connections among producers and consumers contribute to ecosystem structure, function, and stability (e.g., Polis & Strong, 1996; Post, 2002; Worm & Duffy, 2003). Indirect characterization of marine food webs using isotopic analysis of bulk tissues (Rau, 1982) has become routine, particularly for estimating the trophic position (TP) of cetaceans and other marine mammals (Lesage, Hammill, & Kovacs, 2001; Newsome, Clementz, & Koch, 2010). Nitrogen (N) isotope ratios differ by several per mil between food and consumer, and therefore serve as a proxy for TP (DeNiro & Epstein, 1981; McCutchan, Lewis, Kendall, & McGrath, 2003). However, in addition to the suite of dietary (e.g., protein content) and physiological factors (e.g., nutritional state and growth rate) that also influence ^15^N discrimination (Gorokhova, 2018; Nuche‐Pascual, Lazo, Ruiz‐Cooley, & Herzka, 2018; Robbins, Felicetti, & Sponheimer, 2005; Trueman, McGill, & Guyard, 2005), underlying biogeochemical processes impart distinct baseline δ^15^N values to entire food webs (e.g., McClelland, Holl, & Montoya, 2003; Ruiz‐Cooley, Koch, Fiedler, & McCarthy, 2014). Variation in isotopic baselines across a range of spatiotemporal scales can equal or vastly exceed typical trophic ^15^N enrichment (Hannides, Popp, Landry, & Graham, 2009; McMahon, Hamady, & Thorrold, 2013; Rolff, 2000), often leading to the question “do (bulk) nitrogen isotope differences among consumers reflect diet differences or foraging within isotopically distinct food webs?”

The confounding influences of trophic and baseline isotopic variation on bulk tissue SI values can be resolved with concurrent measurement of the isotopic composition of primary producers or available prey. This approach, however, has inherent challenges. The fast growth rates and nutrient uptake of phytoplankton, for example, lead to short‐term isotopic variation that is mismatched with the longer integration periods in consumer tissues (Hannides et al., 2009). Baseline characterization is challenging to resolve for marine mammals that occupy large geographic ranges, and is especially problematic for migratory species whose movements span pronounced regional and seasonally variable isotope gradients. The increasingly popular approach of reconstructing long‐term diets from isotopic profiles of baleen and teeth (Matthews & Ferguson, 2014, 2015; Newsome, Etnier, Monson, & Fogel, 2009; Pomerleau et al., 2018) also introduces temporal baseline SI variation over the period of tissue growth as an additional confounding factor. In such retrospective studies, baseline or prey SI databases over matching temporal scales, which often exceed decades, are typically nonexistent.

Compound‐specific stable isotope analysis (CSIA) of individual amino acids (AAs) offers a means to tease apart trophic and baseline contributions to bulk tissue isotopic variation that circumvents these challenges. Amino acids designated as trophic AAs enter metabolic pathways involving transamination and deamination reactions, during which isotopic discrimination causes ^15^N enrichment of the AA pool (Chikaraishi, Kashiyama, Ogawa, Kitazato, & Ohkouchi, 2007). Source AAs, on the other hand, predominantly enter metabolic pathways in which the amine bonds remain intact, such that primary producer δ^15^N values are conserved with minimal ^15^N enrichment throughout the food web (Chikaraishi et al., 2007, 2009; McClelland & Montoya, 2002). The relative difference between trophic and source AA δ^15^N values of consumer tissues therefore allows for internal calibration of TP while accounting for baseline isotopic variation, making CSIA‐AA ideal for both diet and distribution studies.

Pioneering empirical studies of green algae, zooplankton, and fish larvae measured the relative ^15^N enrichment of multiple AAs with trophic transfer (Chikaraishi et al., 2007, 2009; McClelland & Montoya, 2002). Among source AAs, phenylalanine (Phe) δ^15^N values (δ^15^N_Phe_) were the most conservative, with only a slight increase of ~0.4‰ with each trophic transfer. Glutamic acid + glutamine (Glx; see Methods), on the other hand, exhibited consistent, high ^15^N enrichment of ~8‰ with each trophic transfer (Chikaraishi et al., 2009). It was therefore proposed that consumer TP be calculated as:

(1) $$\text{TP}=\frac{\delta^{15}N_{\text{Glx}}-\delta^{15}N_{\text{Phe}}-\beta_{\text{Glx}\;-\;\text{Phe}}}{{\text{TDF}}_{\text{Glx}\;-\;\text{Phe}}}+1$$

where δ^15^N_Glx_ and δ^15^N_Phe_ are the consumer δ^15^N values of those AAs, β_Glx‐Phe_ is the difference between primary producer δ^15^N_Glx_ and δ^15^N_Phe_ values (3.4 ± 0.9‰ in marine cyanobacteria and algae), and TDF_Glx‐Phe_ is the trophic discrimination factor, or the difference in fractionation of Glx (Δ^15^N_Glx_) and Phe (Δ^15^N_Phe_) with each trophic step (7.6 ± 1.2‰; Chikaraishi et al., 2009). Note that the original publication, along with most other cited ecology studies, uses the abbreviation Glu; here, we use Glx to specify the AAs that are actually measured (see “Methods”).

Equation (1) produces reliable TP estimates for marine invertebrates (Chikaraishi et al., 2009; Hannides et al., 2009) and some fishes (see Choy et al., 2012) and accurately characterizes terrestrial food webs comprising insect and mammal consumers up to TP 5 (Campbell, Nelson, Ogawa, Chikaraishi, & Ohkouchi, 2017; Chikaraishi, Ogawa, Doi, & Ohkouchi, 2011; Chikaraishi et al., 2014; Steffan et al., 2013). However, a growing number of CSIA‐AA studies of higher TP marine consumers like jumbo squid (Ruiz‐Cooley, Ballance, & McCarthy, 2013), elasmobranchs (Dale, Wallsgrove, Popp, & Holland, 2011), tuna (Lorrain et al., 2015), penguins (Lorrain et al., 2009), and killer whales (Matthews & Ferguson, 2014) have reported unrealistically low TP_CSIA_ estimates, indicating TDFs determined empirically for lower TPs cannot be universally applied throughout marine food webs. Controlled feeding studies of large carnivorous fishes (Hoen et al., 2014), penguins (McMahon, Polito, Abel, McCarthy, & Thorrold, 2015), and seals (Germain, Koch, Harvey, & McCarthy, 2013), along with meta‐analyses of a broad range of marine consumer δ^15^N_AA_ values (Bradley et al., 2015; McMahon & McCarthy, 2016; Nielsen, Popp, & Winder, 2015), have since confirmed that the TDF_Glx‐Phe_ in higher consumers is (sometimes substantially) lower than the 7.6‰ measured in invertebrates and fishes. Mechanisms explaining these patterns include the mode of nitrogen excretion (ammonia vs. uric acid or urea) and/or dietary attributes such as protein quantity and composition (Bradley et al., 2015; Chikaraishi, Steffan, Takano, & Ohkouchi, 2015; Germain et al., 2013; Hoen et al., 2014; McMahon, Thorrold, Elsdon, & McCarthy, 2015). Variation in TDF_Glx‐Phe_ among marine consumers has nevertheless been related to TP, although with considerable variation at TP > 3.5 (Nielsen et al., 2015).

Recognizing the potential growth in application of CSIA‐AA in cetacean diet studies (e.g., Matthews & Ferguson, 2014; Pomerleau et al., 2017; Ruiz‐Cooley et al., 2017; Ruiz‐Cooley et al., 2014), we assess TP_CSIA_ estimates of five cetacean species against well‐established stomach content estimates (TP_SC_; Pauly, Trites, Capuli, & Christensen, 1998). The species represent a range of TPs: zooplanktivorous bowhead whales (*Balaena mysticetus*); beluga whales (*Delphinapterus leucas*) and short‐beaked common dolphins (*Delphinus delphis*), which feed on invertebrates and fish; sperm whales (*Physeter macrocephalus*), which feed on squid; and fish‐eating (FE) and marine mammal‐eating (MME) killer whale (*Orcinus orca*) ecotypes. TP_CSIA_ estimates were calculated using equation (1), as well as other equations that incorporate marine mammal (Germain et al., 2013) or seabird‐specific (McMahon, Polito, et al., 2015) TDFs derived from controlled feeding studies. We evaluate primarily whether current TDFs and equations provide accurate TP_CSIA_ estimates, and in the interim absence of accurate TDFs, whether δ^15^N_Glx‐Phe_ is a reliable index of relative TP.

## 2 METHODS

### 2.1 Specimen collection

Bowhead whale skin biopsies (*n* = 10 whales) were collected from free‐ranging animals at Disko Bay, Greenland, using a crossbow. Baleen was collected from subsistence hunted whales (*n* = 2 different whales, from the same population) in the eastern Canadian Arctic. Dolphin skin was collected from animals (*n* = 9) killed incidentally in gillnet fisheries in the Southern California Bight, California, USA. Sperm whale skin was biopsied from free‐ranging and stranded animals (*n* = 13) in the upper California Current. Sperm whale teeth were collected from commercially harvested whales (*n* = 6) off the coast of Peru (Clarke, Paliza, & Aguayo, 1988). Beluga skin (*n* = 4 whales) and teeth (*n* = 9 different whales from the same population) were collected from subsistence hunted animals in the eastern Canadian Arctic. Finally, teeth were collected from genetically assigned FE (*n* = 3) and MME (*n* = 4) killer whale ecotypes stranded around Vancouver Island, Canada (G. Hanke, Royal British Columbia Museum, Pers. Comm.). Tissues were frozen at −20°C with no preservative (bowhead baleen, common dolphin skin, and beluga skin and teeth), frozen at −20°C in 20% dimethyl sulfoxide (DMSO; bowhead and sperm whale skin), or stored dry at room temperature (killer whale teeth).

### 2.2 Sample preparation

Most of the isotope data presented here have been compiled from previously published studies where detailed sample preparation and analysis procedures can be found (Matthews & Ferguson, 2014, 2015; Pomerleau et al., 2017; Ruiz‐Cooley et al., 2014, 2017; Zupcic‐Moore, Ruiz‐Cooley, Paliza, Koch, & McCarthy, 2017). Briefly, baleen samples were drilled from the proximal end of each plate where the most recent growth corresponds to foraging on the summer grounds (Matthews & Ferguson, 2015), and no further sample preparation was carried out prior to isotope analysis. Bowhead whale skin samples were rinsed of DMSO using deionized water and were not lipid‐extracted prior to analysis. Sperm whale skin samples were also rinsed of DMSO using deionized water and then lipid‐extracted using a 2:1 chloroform:ethanol mixture (Lesage et al., 2010; Ruiz‐Cooley, Engelhaupt, & Ortega‐Ortiz, 2012). Dolphin skin was thawed and lipid‐extracted with petroleum ether. Annual dentine growth layers of sperm whale teeth were sampled using a micromill and later combined, while a handheld rotary tool was used to collectively sample multiple dentine growth layers of beluga and killer whale teeth. All dentine was demineralized using repeated washes of 0.25 N HCl for 12‐hr periods, and the remaining collagen was rinsed with distilled H_2_O. All samples except baleen were freeze‐dried and finely homogenized.

### 2.3 Compound‐specific stable isotope analysis

All bowhead, dolphin, beluga, and killer whale tissues were analyzed at the University of California‐Davis Stable Isotope Facility, while sperm whale tissues were analyzed at University of California‐Santa Cruz Stable Isotope Laboratory. Briefly, at UC Davis, approximately 3 mg of each freeze‐dried, homogenized tissue sample was acid‐hydrolyzed using 6 M HCl at 150°C under a N_2_ headspace for 70 min and derivatized using methoxycarbonylation esterification (Walsh, He, & Yarnes, 2014; Yarnes & Herszage, 2017). Methods at UC Santa Cruz differed primarily in the use of trifluoroacyl‐isopropyl ester as the derivatization agent. δ^15^N values of individual derivatized AAs were measured at both laboratories by gas chromatography–combustion isotope ratio mass spectrometry (GC‐IRMS). At UC Davis, two AA mixtures, previously calibrated to the international reference scale for δ^15^N (atmospheric N_2_), were used in calibration and scale‐normalization procedures. Quality assurance of δ^15^N measurements followed Yarnes and Herzsage (2017). A third AA mixture served as the primary quality control reference material, while two well‐characterized natural materials, baleen and fish muscle, were used as secondary quality control standards. At UC Santa Cruz, tissue samples and a control (Cyanno) were hydrolyzed to quantify δ^15^N values from AAs. All derivatives were injected with the AA control, N‐leucine, to verify accuracy during each run. Each sample was run 3–4 times to maximize accuracy among chromatograms. Mean analytical precision assessed from duplicate or triplicate measures of samples and the reference compound was <1‰ at both laboratories.

The suite of AAs that can be accurately quantified depends on the derivatization agent (Ohkouchi et al., 2017) and tissue AA content; we include here the nine AAs that were measured in all samples: Glx, aspartic acid (Asx), alanine (Ala), isoleucine (Ile), leucine (Leu), proline (Pro), valine (Val), glycine (Gly), and Phe. We note that acid hydrolysis converts glutamine (Gln) and asparagine (Asn) to glutamic acid (Glu) and aspartic acid (Asp), respectively. Glx (Glu + Gln) and Asx (Asp + Asn) are the IUPAC‐recognized abbreviations for the resultant AA combinations that are measured.

### 2.4 Trophic position estimates and data analysis

TP_CSIA_ estimates were calculated using four published equations that use the trophic–source AA pair Glx and Phe (Table 1), with the exception of equation (2), which incorporates mean values of multiple source and trophic AAs to minimize the influence of any single AA (Nielsen et al., 2015; Sherwood, Lehmann, Schubert, Scott, & McCarthy, 2011). For equation (2), we used the means of all seven trophic AA identified a priori (Glx, Asx, Ala, Ile, Leu, Pro, and Val), along with δ^15^N_Phe_ values because Gly is no longer considered a reliable source AA (McMahon & McCarthy, 2016), and no other source AA (e.g., methionine) was measured in all samples.

**Table 1:** Empirically derived equations for estimating trophic position (TP) from amino acid‐specific δ^15^N values. Equations (1) and (2) apply a blanket trophic discrimination factor (TDF) to all trophic transfers, while equations (3) and (4) incorporate a dual TDF to account for TDF variation in higher consumers. Note that the original publications use the abbreviation Glu instead of Glx

TP estimating equation	Reference
$\left(1\right)\;\text{TP}=\frac{\delta^{15}N_{\text{Glx}}-\delta^{15}N_{\text{Phe}}-\beta }{{\text{TDF}}_{\text{Glx}\;-\;\text{Phe}}}+1$	Chikaraishi et al. (2007, 2009)
$\left(2\right)\;\text{TP}=\frac{\left(\delta^{15}N_{\Sigma \text{trophic}}-\delta^{15}N_{\Sigma \text{source}}\right)-\beta }{{\text{TDF}}_{\text{Glx}-\text{Phe}}}+1$	
$\left(3\right)\;\text{TP}=\frac{\left(\delta^{15}N_{\left(\text{Glx}\right)}-\delta^{15}N_{\left(\text{Phe}\right)}-{\text{TDF}}_{\left(\text{Glx}\;-\;\text{Phe}\right)\text{seal}}-\beta \right)}{{\text{TDF}}_{\left(\text{Glx}-\text{Phe}\right)\text{plankton}}}+2$	Germain et al. (2013)
$\left(4\right)\;\text{TP}=\frac{\delta^{15}N_{\left(\text{Glx}\right)}-\delta^{15}N_{\left(\text{Phe}\right)}-{\text{TDF}}_{\left(\text{Glx}\;-\;\text{Phe}\right)\text{plankton}}-\beta }{{\text{TDF}}_{\left(\text{Glx}\;-\;\text{Phe}\right)\text{penguin}}}+2$	McMahon, Polito, et al. (2015)

Equations (1) and (2), based on the work of McClelland and Montoya (2002) and Chikaraishi et al. (2007, 2009), apply a single TDF_Glx‐Phe_ of 7.6‰ to all trophic transfers, while equations (3) and (4) apply a dual TDF_Glx‐Phe_ to account for variation among trophic transfers. Equation (3) was derived from a controlled feeding study of harbor seals (*Phoca vitulina*) by Germain et al. (2013), who calculated a TDF_Glx‐Phe_ between the seals and their food (herring) of 4.3 ± 1.2‰. Their equation applies the seal‐specific value of 4.3‰ only to the final transfer to the seal. Equation (4) was derived by McMahon, Polito, et al. (2015), who calculated a TDF_Glx‐Phe_ of 3.5 ± 0.4‰ in captive gentoo penguins (*Pygoscelis papua*) on a controlled diet. It differs from equation (3) in its application of the lower, penguin‐specific TDF to all trophic transfers after the first one between algae and zooplankton. We include this equation because, like many cetaceans, penguins occupy high TPs and have diets comprising fish and squid. Moreover, as urea and uric acid excreters, respectively, cetaceans and penguins are expected to have comparably low TDF_Glx‐Phe_ values relative to ammonia excreters (McMahon & McCarthy, 2016).

In all equations, δ^15^N_Glx_ and δ^15^N_Phe_ are the measured consumer δ^15^N values of those AAs, β is the δ^15^N difference between δ^15^N_Glx_ and δ^15^N_Phe_ in primary producers (3.4‰ for algae and cyanobacteria; McClelland & Montoya, 2002), and TDF_Glx‐Phe_ (equation 1) and TDF_(Glx‐Phe) plankton_ (equations 3 and 4) are the relative difference between δ^15^N_Glx_ and δ^15^N_Phe_ with each trophic transfer derived from experimental studies on invertebrates and fish (7.6‰). Uncertainty in TDF and β estimates and δ^15^N_AA_ measurements was propagated through to final TP_CSIA_ estimates using first‐order Taylor series expansion in the R (version 3.1.3; R Core team, 2015) package “propagate” (Spiess, 2018). Errors used for the various TDFs and β are published in Chikaraishi et al. (2009), Germain et al. (2013), and McMahon, Polito, et al. (2015), while tissue‐specific errors in δ^15^N_Glx_ and δ^15^N_Phe_ were calculated from duplicate or triplicate sample measurements. TP_CSIA_ estimates were qualitatively assessed against TP_SC_ estimates calculated for each species based on multiple studies (Pauly et al., 1998).

## 3 RESULTS

**Figure 1:**
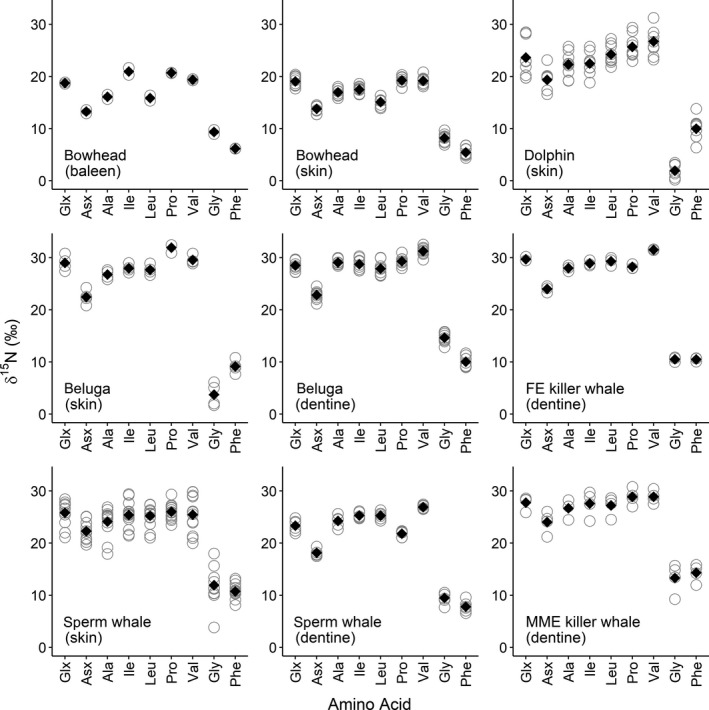
Mean (solid diamonds) and individual (hollow circles) δ^15^N measurements of nine amino acids (AAs) in tissues (baleen, skin, and/or dentine) of five cetacean species

δ^15^N values of individual trophic AAs (Glx, Asx, Ala, Ile, Leu, Pro, and Val) ranged from 12.93 to 33.73‰ across all samples. Within species, bowhead whales generally had the lowest mean trophic AA δ^15^N values (13.26–20.95‰) compared to the rest, which generally ranged from the low 20s to low 30s (Table 2, Figure 1). Among trophic AA, either Pro or Val δ^15^N values were highest in all species and tissues, while Asx δ^15^N values were always lowest (Table 2, Figure 1). Glx δ^15^N was variable among species, with the lowest values in bowhead whales and the highest in FE killer whales. All trophic AAs were ^15^N‐enriched relative to Phe by approximately 10–20‰, and trophic AA δ^15^N values were generally similar between tissues of species for which more than one tissue was measured (Table 2, Figure 1).

**Table 2:** δ^15^N values of nine amino acids in tissues of five cetacean species (mean ± standard deviation)

Species	Tissue	Glutamic acid (Glx)	Aspartic acid (Asx)	Alanine	Isoleucine	Leucine	Proline	Valine	Glycine	Phenylalanine
Bowhead whale (*Balaena mysticetus*)	Baleen (*n* = 2)	18.75	13.26	16.11	20.95	15.86	20.70	19.39	9.36	6.17
	Skin (*n* = 10)	19.02 ± 0.9	13.91 ± 0.7	17.03 ± 0.8	17.42 ± 0.7	15.24 ± 0.9	19.41 ± 0.7	19.29 ± 0.8	8.18 ± 0.8	5.53 ± 0.9
Beluga (*Delphinapterus leucas*)	Skin (*n* = 4)	28.99 ± 1.4	22.45 ± 1.4	26.77 ± 0.8	27.95 ± 0.8	27.65 ± 0.9	32.37 ± 1.2	29.55 ± 0.9	3.73 ± 2.2	9.14 ± 1.3
	Dentine collagen (*n* = 9)	28.49 ± 1.0	22.84 ± 0.9	29.07 ± 0.5	28.75 ± 1.1	27.89 ± 1.3	29.30 ± 0.8	31.25 ± 0.8	14.64 ± 1.0	10.04 ± 1.0
Common dolphin (*Delphinus delphis*)	Skin (*n* = 9)	23.62 ± 3.8	19.35 ± 2.0	22.30 ± 2.3	22.47 ± 2.1	24.25 ± 1.8	25.67 ± 2.2	26.72 ± 2.5	0.84 ± 2.6	9.97 ± 2.1
FE killer whale (*Orcinus orca*)	Dentine collagen (*n* = 3)	29.69 ± 0.4	24.00 ± 0.6	27.99 ± 0.6	28.90 ± 0.5	29.30 ± 0.8	28.24 ± 0.5	32.04 ± 0.9	10.52 ± 0.5	10.48 ± 0.4
Sperm whale (*Physeter macrocephalus*)	Skin (*n* = 13)	25.81 ± 2.2	22.29 ± 2.0	24.13 ± 2.7	25.39 ± 2.5	25.25 ± 2.1	26.04 ± 1.5	25.46 ± 3.5	11.93 ± 3.5	10.74 ± 1.5
	Dentine collagen (*n* = 6)	23.33 ± 1.1	18.13 ± 0.6	24.25 ± 1.0	25.28 ± 0.6	25.28 ± 0.7	21.80 ± 0.6	26.92 ± 0.4	9.48 ± 1.0	7.82 ± 1.1
MME killer whale (*Orcinus orca*)	Dentine collagen (*n* = 4)	27.77 ± 1.3	24.03 ± 2.0	26.69 ± 1.6	27.58 ± 2.4	27.23 ± 1.9	28.89 ± 1.6	28.88 ± 1.2	13.31 ± 2.8	14.31 ± 1.7

Abbreviations: FE, fish‐eating; MME, marine mammal‐eating.

Among AAs currently or previously classified as source AAs, mean Phe and Gly δ^15^N values were similar among species and tissues, with the exception of greater Phe values in beluga and dolphin skin (by ~5 to 9‰, respectively; Table 2). Mean Phe δ^15^N values ranged from 5.53 to 14.31‰ across all species and were generally lowest in bowhead whales (means for skin and baleen and were 5.53 and 6.17‰, respectively) compared to the other species (7.82‰ in sperm whale dentine to 14.31‰ in MME killer whales; Table 2). Notably, Phe δ^15^N values were ~4‰ higher in MME (14.31‰) than FE (10.48‰) killer whales. δ^15^N values of Phe and Gly were similar between bowhead whale baleen and skin, but differed between skin and dentine collagen of beluga and sperm whales. Gly in particular differed by more than 10‰ (Table 2 and Figure 1).

TP_CSIA_ estimates using all equations were lower than published TP_SC_ (Table 3). Equations (1) and (2) produced TP_CSIA_ estimates that were generally 1 to 2.5 positions lower than TP_SC_ for all species (Table 3). Equations (3) and (4), which apply dual TDFs to account for lower TDF in higher consumers, produced TP_CSIA_ estimates that were generally comparable to TP_SC_ for bowhead whales, belugas, and FE killer whales, but 0.5–1.5 positions lower than TP_SC_ for dolphins and sperm whales, and 2–2.5 lower for MME killer whales (Table 3). Tissue‐specific TP_CSIA_ estimates were similar within species (Table 3).

**Table 3:** Amino acid δ^15^N‐derived trophic position (TP) estimates for five cetacean species compared against those established from stomach contents (TP_SC_) from Pauly et al. (1998). TP estimates (1–4) were calculated using published equations (Table 1) from Chikaraishi et al. (2009) (equations 1 and 2), Germain et al. (2013) (equation 3), and McMahon, Polito, et al. (2015) (equation 4). Each row represents an individual whale, and errors in estimates of equation parameters (TDF and ß) and δ^15^N_AA_ measurements were propagated to produce errors around the TP estimates (see Methods)

Species	Tissue	δ^15^N_Glx‐Phe_ (‰)	TP_(SC)_	TP (1)	TP (2)	TP (3)	TP (4)
Bowhead whale (*Balaena mysticetus*)	Baleen (*n* = 2)	12.40	3.2	2.2 ± 0.3	1.9 ± 0.3	2.6 ± 0.3	2.4 ± 0.6
		12.75		2.2 ± 0.3	1.9 ± 0.3	2.7 ± 0.3	2.5 ± 0.6
	Skin (*n* = 10)	14.47		2.5 ± 0.3	1.9 ± 0.2	2.9 ± 0.3	3.0 ± 0.5
		12.63		2.2 ± 0.2	1.9 ± 0.2	2.6 ± 0.2	2.5 ± 0.5
		12.68		2.2 ± 0.2	1.9 ± 0.3	2.7 ± 0.2	2.5 ± 0.5
		13.57		2.3 ± 0.3	1.9 ± 0.2	2.8 ± 0.2	2.7 ± 0.5
		13.82		2.4 ± 0.3	2.0 ± 0.2	2.8 ± 0.3	2.8 ± 0.5
		13.28		2.3 ± 0.3	2.0 ± 0.2	2.7 ± 0.2	2.7 ± 0.5
		13.31		2.3 ± 0.3	1.9 ± 0.2	2.7 ± 0.2	2.7 ± 0.5
		13.84		2.4 ± 0.3	2.0 ± 0.2	2.8 ± 0.3	2.8 ± 0.5
		14.49		2.5 ± 0.3	1.9 ± 0.2	2.9 ± 0.3	3.0 ± 0.5
		13.84		2.4 ± 0.3	2.0 ± 0.3	2.8 ± 0.3	2.8 ± 0.5
Beluga (*Delphinapterus leucas*)	Skin (*n* = 4)	20.16	4.0	3.2 ± 0.4	3.2 ± 0.4	3.6 ± 0.3	4.6 ± 0.6
		19.46		3.1 ± 0.4	3.5 ± 0.4	3.6 ± 0.3	4.4 ± 0.5
		19.76		3.2 ± 0.4	3.4 ± 0.4	3.6 ± 0.3	4.5 ± 0.6
		20.01		3.2 ± 0.4	3.4 ± 0.4	3.6 ± 0.3	4.6 ± 0.6
	Dentine collagen (*n* = 9)	17.10		2.8 ± 0.3	2.6 ± 0.3	3.2 ± 0.3	3.7 ± 0.5
		18.64		3.0 ± 0.4	2.7 ± 0.3	3.4 ± 0.3	4.2 ± 0.5
		18.17		2.9 ± 0.3	2.7 ± 0.3	3.4 ± 0.3	4.1 ± 0.5
		18.97		3.0 ± 0.4	2.5 ± 0.3	3.5 ± 0.3	4.3 ± 0.6
		20.73		3.3 ± 0.4	2.7 ± 0. 3	3.7 ± 0.4	4.8 ± 0.6
		16.91		2.8 ± 0.3	2.6 ± 0.3	3.2 ± 0.3	3.7 ± 0.5
		18.88		3.0 ± 0.4	2.7 ± 0.3	3.5 ± 0.3	4.3 ± 0.5
		18.54		3.0 ± 0.4	2.6 ± 0.3	3.4 ± 0.3	4.2 ± 0.5
		18.14		2.9 ± 0.3	2.7 ± 0.3	3.4 ± 0.3	4.0 ± 0.5
Common dolphin (*Delphinus delphis*)	Skin (*n* = 9)	11.20	4.2	2.0 ± 0.2	3.1 ± 0.4	2.5 ± 0.2	2.1 ± 0.5
		13.87		2.4 ± 0.3	2.8 ± 0.3	2.8 ± 0.3	2.8 ± 0.5
		11.28		2.0 ± 0.2	3.1 ± 0.4	2.5 ± 0.2	2.1 ± 0.5
		9.23		1.8 ± 0.2	2.8 ± 0.3	2.2 ± 0.2	1.5 ± 0.5
		13.22		2.3 ± 0.3	3.0 ± 0.3	2.7 ± 0.2	2.6 ± 0.5
		17.55		2.9 ± 0.3	3.0 ± 0.3	3.3 ± 0.3	3.9 ± 0.5
		11.24		2.0 ± 0.2	2.7 ± 0.3	2.5 ± 0.2	2.1 ± 0.5
		18.02		2.9 ± 0.3	3.0 ± 0.4	3.4 ± 0.3	4.0 ± 0.5
		17.22		2.8 ± 0.3	2.9 ± 0.3	3.3 ± 0.3	3.8 ± 0.5
FE killer whale (*Orcinus orca*)	Dentine collagen (*n* = 3)	19.50	4.3^a^	3.1 ± 0.4	2.9 ± 0.3	3.6 ± 0.3	4.4 ± 0.6
		18.71		3.0 ± 0.4	2.9 ± 0.3	3.5 ± 0.3	4.2 ± 0.5
		19.40		3.1 ± 0.4	2.9 ± 0.3	3.5 ± 0.3	4.4 ± 0.6
Sperm whale (*Physeter macrocephalus*)	Skin (*n* = 13)	15.16	4.4	2.6 ± 0.3	2.2 ± 0.2	3.0 ± 0.3	3.2 ± 0.5
		16.36		2.7 ± 0.3	2.2 ± 0.2	3.1 ± 0.3	3.5 ± 0.5
		15.27		2.6 ± 0.3	2.2 ± 0.2	3.0 ± 0.3	3.2 ± 0.5
		17.73		2.9 ± 0.3	2.6 ± 0.3	3.3 ± 0.3	3.9 ± 0.5
		14.97		2.5 ± 0.3	2.0 ± 0.2	3.0 ± 0.3	3.1 ± 0.5
		15.15		2.6 ± 0.3	2.3 ± 0.3	3.0 ± 0.3	3.2 ± 0.5
		14.99		2.5 ± 0.3	2.0 ± 0.2	3.0 ± 0.3	3.1 ± 0.5
		11.95		2.1 ± 0.2	1.6 ± 0.2	2.6 ± 0.2	2.3 ± 0.5
		12.64		2.2 ± 0.2	1.9 ± 0.2	2.7 ± 0.2	2.5 ± 0.5
		18.57		3.0 ± 0.3	3.0 ± 0.4	3.4 ± 0.3	4.2 ± 0.5
		13.62		2.3 ± 0.3	2.0 ± 0.2	2.8 ± 0.3	2.8 ± 0.5
		16.67		2.8 ± 0.3	2.5 ± 0.3	3.2 ± 0.3	3.6 ± 0.5
		12.84		2.2 ± 0.3	2.1 ± 0.2	2.7 ± 0.2	2.5 ± 0.5
	Dentine collagen (*n* = 6)	15.30		2.6 ± 0.3	2.6 ± 0.3	3.0 ± 0.3	3.2 ± 0.5
		15.80		2.6 ± 0.3	2.6 ± 0.3	3.1 ± 0.3	3.4 ± 0.5
		16.10		2.7 ± 0.3	2.5 ± 0.3	3.1 ± 0.3	3.5 ± 0.5
		13.60		2.3 ± 0.3	2.5 ± 0.3	2.8 ± 0.3	2.7 ± 0.5
		17.10		2.8 ± 0.3	2.5 ± 0.3	3.2 ± 0.3	3.7 ± 0.5
		15.20		2.6 ± 0.3	2.4 ± 0.3	3.0 ± 0.3	3.2 ± 0.5
MME killer whale (*Orcinus orca*)	Dentine collagen (*n* = 4)	13.36	5.0^a^	2.3 ± 0.3	2.2 ± 0.2	2.7 ± 0.3	2.7 ± 0.5
		13.95		2.4 ± 0.3	2.4 ± 0.3	2.8 ± 0.3	2.8 ± 0.5
		12.51		2.2 ± 0.2	2.4 ± 0.3	2.6 ± 0.2	2.4 ± 0.5
		14.03		2.4 ± 0.3	2.3 ± 0.3	2.8 ± 0.3	2.9 ± 0.5

Abbreviations: FE, fish‐eating; MME, marine mammal‐eating.

^a^ Pauly et al. (1998) calculated one value for killer whales (4.5), assuming approximately equal proportions of miscellaneous fishes and higher vertebrates, and lesser amounts of squids and pelagic fishes. Following their methodology, we calculated a FE killer whale TP assuming diet comprised 100% salmon (Ford & Ellis, 2006), which Pauly et al. (1998) assigned a TP of 3.3, and MME killer whale TP assuming diet comprised 100% higher vertebrates, which Pauly et al. (1998) assigned a value of 4.0. The mean trophic positions for each prey type are originally from Pauly and Christensen (1995).

**Figure 2:**
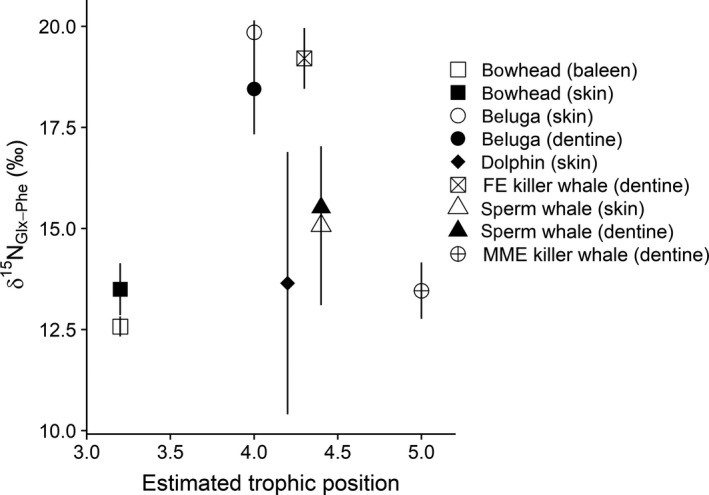
δ^15^N_Glx‐Phe_ values in five cetacean species plotted against their estimated trophic position from stomach contents (Pauly et al., 1998). The lack of relationship between δ^15^N_Glx‐Phe_ and trophic position (predicted to be positively correlated) indicates δ^15^N_Glx‐Phe_ is not a reliable proxy for relative trophic position in these species

**Figure 3:**
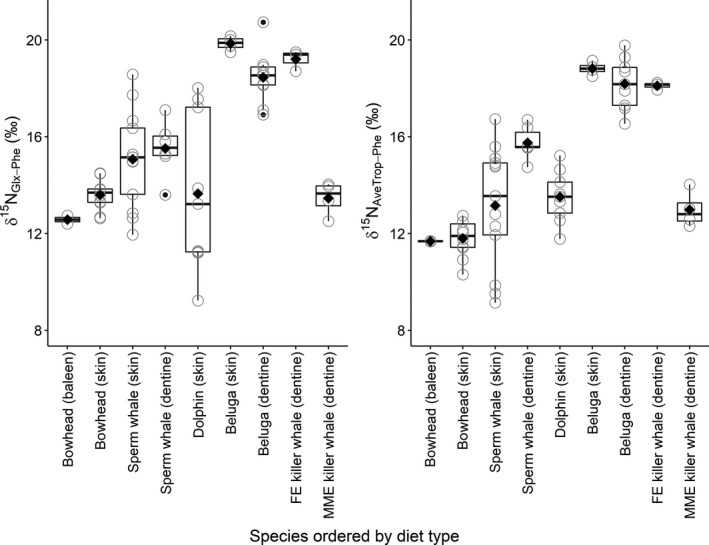
δ^15^N_Glx‐Phe_ (left panel) and δ^15^N_AveTrop‐Phe_ (right) values in five cetacean species ordered by diet type (zooplankton eating bowhead whales, squid‐eating sperm whales, fish/invertebrate eating dolphin and beluga whales, fish‐eating killer whales, and marine mammal‐eating killer whales)

Mean δ^15^N_Glx‐Phe_ values did not follow TP_SC_ order. The lowest values were in bowhead whales (12.57 and 13.50‰ in baleen and skin, respectively) and, notably, MME killer whales (13.46‰; Table 3; Figures 2 and 3). The highest mean δ^15^N_Glx‐Phe_ values were in beluga (19.85‰ and 18.45‰ in skin and dentine collagen, respectively) and FE killer whales (19.21‰; Table 3; Figures 2 and 3). Mean δ^15^N_Glx‐Phe_ was 13.65‰ in common dolphins, which exhibited highly variable values among individuals compared to the other species. Sperm whales had intermediate mean δ^15^N_Glx‐Phe_ values relative to the other species, 15.07‰ and 15.52‰ in skin and dentine collagen, respectively (Table 2; Figures 2 and 3).

## 4 DISCUSSION

This study presents the most comprehensive compilation of AA δ^15^N values and TP_CSIA_ estimates for cetaceans, which have been underrepresented in recent meta‐analyses of marine consumer CSIA‐AA due to lack of published data, and for which no published controlled diet studies exist. Our findings are consistent with general patterns across a range of other taxa from diverse marine ecosystems that have shown variable AA isotopic fractionation (Bradley et al., 2015; McMahon & McCarthy, 2016; Nielsen et al., 2015) and low TP_CSIA_ estimates for higher TP consumers (Dale et al., 2011; Lorrain et al., 2009, 2015; Matthews & Ferguson, 2014; Ruiz‐Cooley et al., 2013).

We anticipated lower TP_CSIA_ estimates assuming a uniform TDF across all trophic transfers (Equations 1 and 2), given previous studies have shown that TDF_Glx‐Phe_ values in other high TP consumers like harbor seals (Germain et al., 2013) and penguins (McMahon, Polito, et al., 2015) were considerably lower than those measured in invertebrates and fish (Chikaraishi et al., 2007, 2009; McClelland & Montoya, 2002). Equation (1) assumes that δ^15^N_Glx_ values are the highest among trophic AAs, which was not the case in any cetacean tissue. Other studies have also found slightly lower TDF_Glx_ values relative to other trophic AAs such as Pro (e.g., Bradley et al., 2015). Higher δ^15^N_Pro_ values in many of our samples might reflect its role in formation of collagen (Germain et al., 2013), a prominent protein in skin and dentine. Averaging across the δ^15^N values of all trophic AAs using equation (2), which is intended to minimize the impact of such δ^15^N variation in any single AA, did not improve TP estimates, possibly because higher δ^15^N values of Pro and Val were offset by the relatively low δ^15^N_Asx_. Other studies have similarly shown no relationship between δ^15^N_Asx_ and TP in marine consumers (Bradley et al., 2015). Although alternative trophic and source AAs have been suggested (e.g., Pro [McMahon, Thorrold, et al., 2015]), none of the trophic δ^15^N_AA_ values differed from δ^15^N_Glx_ to an extent that would alter resultant TP_CSIA_ estimates; unfortunately, methionine could not be measured across all samples and its suitability as an alternative source AA cannot be evaluated here.

TP_CSIA_ estimates using the dual TDF approach, which has been advocated to account for variation in trophic ^15^N enrichment across TPs, were still often more than one TP lower than TP_SC_. The low TP_CSIA_ estimates for all species using equation (3), which applies a smaller TDF of 4.3‰ to just the final trophic transfer (Germain et al., 2013), could possibly reflect differences in AA metabolism between the captive seals and wild cetaceans (driven, e.g., by rate and amount of food intake, protein content, metabolic processing, etc.). However, Germain et al.’s (2013) own TP_CSIA_ estimate of 2.8 for harbor seals from which the equation was derived is also unrealistically low for seals fed wild‐caught herring. Herring is a secondary consumer of zooplankton that itself occupies at a TP ~ 3 (Pauly & Christensen, 1995), thus putting the seals at an expected TP of ~4.

TP_CSIA_ estimates using the dual TDF equation (4) of McMahon, Polito, et al. (2015) were in better agreement with TP_SC_ estimates for bowhead whales, belugas, and FE killer whales, but underestimated TP_SC_ for common dolphins, sperm whales, and MME killer whales by more than 1. The fundamental difference between this and Germain et al.’s (2013) equation is the placement of the lower penguin‐specific TDF in the equation's denominator, which applies the smaller TDF to all trophic transfers beyond algae and zooplankton. While this is at odds with studies that have measured a TDF of 7.6‰ between zooplankton and fish (Chikaraishi et al., 2009), subsequent studies have shown higher TDFs in herbivores (~6 to 8‰) than omnivores and carnivores (TDF < 7.6‰; Hoen et al., 2014; McMahon & McCarthy, 2016; Nielsen et al., 2015), which justifies application of a lower TDF to trophic transfers after primary consumers.

Improved TP_CSIA_ estimates using equation (4) support the emerging consensus that applying a single, universal TDF across all TPs is inappropriate and is also consistent with the inverse relationship between bulk tissue ^15^N fractionation and TP in marine consumers (Hussey et al., 2013). However, the low and inconsistent TP_CSIA_ estimates for sperm whales, dolphins, and especially MME killer whales indicate that TDFs derived from a marine mammal (seal) and seabird (penguin) cannot be broadly applied across upper TP marine mammal consumers. Others have suggested TDFs might be taxon‐specific or related to excretion type, diet composition, and/or TP (Hoen et al., 2014; McMahon & McCarthy, 2016; Nuche‐Pascual et al., 2018). Applying multiple TDFs to account for such variation with diet composition and/or TP would require significant a priori knowledge of an animal's diet to determine both the number and values of TDFs that need to be applied, a level of detail, which, if available, defeats the purpose of applying CSIA‐AA to characterize TP. This is illustrated, for example, by the particular case of FE and MME killer whales and is also relevant for other cetaceans and pinnipeds that forage at least occasionally on other marine mammals (e.g., false [*Pseudorca crassidens*] and pygmy [*Feresa attenuata*] killer whales; walruses [*Odobenus rosmarus*]; Pauly et al., 1998). Use of CSIA‐AA will therefore require models that incorporate such complexities of AA fractionation, and may benefit from further study of threonine (Thr), an AA that apparently has constant ^15^N *depletion* with trophic level (Bradley et al., 2015), and may therefore be more appropriate for TP reconstructions (Fuller & Petzke, 2017). Unfortunately, Thr was not measured across all samples in this study and therefore cannot be evaluated here.

Baleen, skin, and dentine are composed of different proteins, and their unique AA compositions and metabolic rates may impart tissue‐specific ^15^N fractionation as each tissue draws differentially on AA pools during formation (see Schmidt et al., 2004). Sampled tissues were not from the same animals, preventing direct comparison of tissue‐specific δ^15^N_AA_ values. Relative patterns of variation for trophic AAs were nevertheless largely similar between tissues and across species, suggesting that the metabolic processes driving their isotopic fractionation follow similar biochemical pathways in cetaceans. A notable exception was Gly, which had considerably lower δ^15^N values (~10‰) relative to Phe in beluga and dolphin skin, but not in bowhead and sperm whale skin. Skin from by‐caught dolphins and hunted belugas that were potentially molting may have been subjected to some degree of bacterial degradation, as opposed to freshly biopsied bowhead and sperm whale skin. Calleja, Batista, Peacock, Kudela, and McCarthy (2013), however, showed bacterially degraded organic nitrogen had approximately 15‰ higher δ^15^N_Gly_ values than fresh material, which is inconsistent with the lower values we observed in skin. Baleen, skin, and teeth are routinely sampled from cetaceans during field research programs and necropsies, and tissue‐specific AA‐specific δ^15^N variation merits further study (preferably using multiple tissues sampled from the same individuals) to understand how tissue selection might impact TP_CSIA_ estimation.

Some degree of discrepancy between TP_CSIA_ and TP_SC_ estimates could reflect inaccurate diet assumptions, as stomach contents may be biased toward recent diet and items with differential digestion rates (Bowen & Iverson, 2013). However, we have included species whose diets have been well characterized through meta‐analysis of numerous studies (Pauly et al., 1998). Population‐specific and individual diet differences can be considerable, but not to the degree required to make sense of TP_CSIA_ estimates that are off by 1–2 TPs. Variation in ß_Glx‐Phe_ (see Vander Zanden et al., 2013) would also lead to erroneous TP_CSIA_ estimates using equations that assume a constant ß across different marine food webs. Back‐calculated estimates of ß from regression analyses of hundreds of marine consumer δ^15^N_AA_ values (Bradley et al., 2015; Nielsen et al., 2015), however, are consistent with the value used in our calculations (Chikaraishi et al., 2009). ß values of sea grasses and terrestrial C3 plants can be more than 10‰ lower than marine algae (Chikaraishi et al., 2009; Vander Zanden et al., 2013), which could explain the low TP_CSIA_ estimates of dolphins feeding in coastal food webs with sea grass or allochthonous terrestrial inputs (see Barros, Ostrom, Stricker, & Wells, 2010). While we know of no measured ß values in ice algae, which contribute considerably to the Arctic food webs of bowhead whales and belugas (Brown et al., 2017), TP_CSIA_ estimates were no worse for these species than the others.

Ohkouchi et al. (2017) and others (e.g., McMahon & McCarthy, 2016) have noted that derivatization via methoxycarbonylation esterification, used for the majority of samples in this study, can be problematic for Glx measurements, given pH‐dependent fractionation can produce two Glu derivatives with distinct δ^15^N values. In fact, at pH < 1, methoxycarbonylation esterification produces one primary derivatization product (pyroglutamic acid) that retains the δ^15^N value of the original glutamic acid (Walsh et al., 2014; Yarnes & Herszage, 2017). The use of a derivatization medium of 0.4 M HCl and constant monitoring ensured all analyses were conducted well under pH 1, and chromatograms did not exhibit a secondary peak. Moreover, derivatization of killer whale dentine samples (*n* = 9) using methoxycarbonylation esterification and two other common derivatization methods, *N*‐acetylation–isopropylation and trifluoroacyl‐isopropyl esterification, produced similar Glx (as well as Phe) δ^15^N values (Matthews & Ferguson, 2014; Yarnes & Herszage, 2017). We therefore conclude that our low TP_CSIA_ estimates are not an artifact of derivatization method, which is supported by the similar results for sperm whale samples derivatized using trifluoroacyl‐isopropyl esterification. This argument, however, will be unavoidable until international standards are used for interlaboratory CSIA‐AA calibration, as is routinely done with bulk SIA.

In the absence of accurate TDFs and estimating equations, we had anticipated that δ^15^N_Glx‐Phe_ would at least serve as an index of *relative* TP; that is, the relative difference in δ^15^N_Glx‐Phe_ among consumers would follow TP_SC_ order. Nonsystematic differences in δ^15^N_Glx‐Phe_ with respect to TP_SC_ are nevertheless consistent with recent meta‐analyses that, despite finding significant overall positive correlations with TP, report considerable variation in δ^15^N_Glx‐Phe_ spanning 10‰ in higher consumers (Bradley et al., 2015; Nielsen et al., 2015). McMahon and McCarthy (2016) found TDF_Glx‐Phe_ values across 70 species varied predominantly with mode of N excretion and diet quality. Because our analysis focused on a single infraorder, mode of N excretion cannot account for the observed δ^15^N_Glx‐Phe_ variation. Reorganizing species based broadly on diet type (Figure 3), however, shows δ^15^N_Glx‐Phe_ differences may reflect diet quality differences. Zooplanktivorous bowhead whales had the lowest δ^15^N_Glx‐Phe_, followed by offshore, primarily mesopelagic squid‐eating sperm whales, and then fish‐eating belugas and FE killer whales, a pattern that is consistent with the increasing deviation of TP_CSIA_ with mean TP of feeding guild (invertivores vs. piscivores) of carnivorous fishes (Bradley et al., 2015). Incorporation of N derived from the foregut fermentation of chitin exoskeletons of crustacean prey (Herwig, Staley, Nerini, & Braham, 1984; Sanders et al., 2015) may have contributed to the δ^15^N_Glx‐Phe_ of bowhead whales, the only mysticete in our sample, although the relative importance of this potential influence is unknown. The lower (and considerably more variable) δ^15^N_Glx‐Phe_ values of common dolphins, which feed near the coast on small sized fish (mainly myctophids) and cephalopods, could reflect different primary producer inputs (see above), or prey assemblages from different length food chains related to temporal variation in environmental conditions (see Ruiz‐Cooley et al., 2017).

The most surprising divergence from any apparent relationship between δ^15^N_Glx‐Phe_ and diet type was the unexpectedly low δ^15^N_Glx‐Phe_ of MME killer whales (Figure 3). The fish diets of belugas and FE killer whales and marine mammal diets of MME killer whales would presumably both be high in protein content, and Beach et al. (1943) report similar proportions of 10 AAs in muscle of fish and mammals, suggesting they would be of similar quality (i.e., AA composition). However, it is possible that dietary AA imbalances (e.g., amounts present in the diet vs. those required for growth and metabolism) might be larger for whales feeding on fish than those feeding on other marine mammals, thereby leading to differences in isotopic fractionation (see Martínez del Río, Wolf, Carleton, & Gannes, 2009; Robbins et al., 2005). Proportional protein content in the diets of the two killer whale ecotypes could also differ, particularly if MME killer whales consume higher amounts of lipids via blubber (see Browning[, Dold, I‐Fan, & Worthy, 2014], who found Δ^15^N_bulk_ values in dolphin skin [*Tursiops truncatus*] varied with lipid content of diet).

Studies regarding the impact of protein content and quality and AA imbalance on AA‐specific δ^15^N fractionation have provided contrasting results. McMahon, Thorrold, et al. (2015) found fish consumers (*Fundulus heteroclitus*) fed high‐protein‐content diets with similar AA compositions to their own tissues had significantly lower TDF_Glx‐Phe_ values than fish fed low‐protein‐quantity and low‐protein‐quality (plant‐based) diets. In contrast, Chikaraishi et al. (2015) found tadpoles (*Bufo japonicus*) fed high‐quality diets with presumably adequate protein content had TDF_Glx_ values that were significantly higher than tadpoles fed a protein‐poor diet. Like previous studies attributing “compressed” TDF_Glx‐Phe_ in higher TP consumers to mechanisms affecting trophic ^15^N enrichment of Glu (Chikaraishi et al., 2015; Germain et al., 2013; McMahon & McCarthy, 2016; McMahon, Thorrold, et al., 2015), both of these studies linked variation in TDF_Glx‐Phe_ to variation in ^15^N fractionation of Glu, since δ^15^N_Phe_ values were essentially constant among treatments.

Few studies have suggested ^15^N enrichment of Phe as a contributing factor to variation in δ^15^N_Glx‐Phe_ with TP, since Phe is assumed to undergo negligible trophic ^15^N enrichment (Bradley et al., 2015; Chikaraishi et al., 2009; McMahon & McCarthy, 2016; Nielsen et al., 2015). However, Nuche‐Pascual et al. (2018) found TEF_Phe_ in muscle of Pacific yellowtail (*Seriola lalandi*) varied by up to 3.3‰ with protein content and quality under controlled diet conditions. The authors suggested variation in trophic ^15^N enrichment of phenylalanine related to diet–consumer AA profiles may reflect the extent to which Phe is used as an energy source versus channeled to growth (Nuche‐Pascual et al., 2018). Our data suggest trophic ^15^N enrichment of Phe should be considered a potential contributing factor to “compressed” TDF_Glx‐Phe_ in higher TP consumers, given whales occupying higher TPs had higher δ^15^N_Phe_ values in three different geographic regions. δ^15^N_Phe_ values of MME killer whales (14.31 ± 1.7‰) were greater than those of sympatric FE killer whales (10.48 ± 0.38‰) in the eastern North Pacific. Finally, δ^15^N_Phe_ values in skin of belugas (9.14 ± 1.3‰) were considerably higher than those of bowhead whale baleen grown in summer (mean 6.17‰), when both species share a similar distribution. Similarly, δ^15^N_Phe_ values of common dolphins (9.97 ± 2.1‰) and sperm whales (10.74 ± 1.5‰) off the California coast were considerably higher than those of lanternfish (Myctophidae) from the same region (~4 to 6‰; Choy et al., 2012).

The large geographic ranges of these species introduce potential for integration of spatially and seasonally variable baseline SI values that precludes any rigorous conclusions regarding trophic ^15^N enrichment of Phe. The only published controlled diet study on marine mammals, harbor seals, does not support this hypothesis (Germain et al., 2013). However, controlled diet studies of rats (Fuller & Petzke, 2017) and fish (Nuche‐Pascual et al., 2018) and meta‐analyses of a broad range of consumer δ^15^N_Phe_ measurements (Bradley et al., 2015; Hoen et al., 2014) revealed variable and sometimes considerable trophic ^15^N fractionation of Phe that ranged from 1 to 3‰, and was greater by nearly 1‰ in upper versus lower TP consumers (McMahon & McCarthy, 2016). When compounded over trophic steps up to TP 5, such increases in δ^15^N_Phe_ could account for much of the deviation between TP_CSIA_ and TP_SC_. Negligible trophic ^15^N enrichment of Phe is a central assumption of CSIA‐AA applications in *both diet and distribution* studies and is *the* factor that allows for confounding trophic and baseline influences to be teased apart by assuming source AA δ^15^N is a proxy for baseline values. Our data indicate that this assumption might be violated and deserves further study under controlled diet settings before CSIA‐AA is routinely applied in ecological (as well as physiological) studies of cetaceans and other higher TP marine consumers.

## CONFLICT OF INTEREST

None declared.

## AUTHORS’ CONTRIBUTIONS

CJDM, RIR‐C, CP, and SHF conceived the ideas and designed methodology. CJDM, RIR‐C, and CP collected and analyzed the data. CJDM and RIR‐C wrote the manuscript. All authors contributed critically to the drafts and gave final approval for publication.

## Data Availability

All data analyzed in this paper have been uploaded to the Dryad Digital Repository (https://doi.org/10.5061/dryad.9kd51c5d3).
